# Inertial Sensor-Based Motion Analysis of Lower Limbs for Rehabilitation Treatments

**DOI:** 10.1155/2017/1949170

**Published:** 2017-07-05

**Authors:** Tongyang Sun, Hua Li, Quanquan Liu, Lihong Duan, Meng Li, Chunbao Wang, Qihong Liu, Weiguang Li, Wanfeng Shang, Zhengzhi Wu, Yulong Wang

**Affiliations:** ^1^School of Mechanical & Automative Engineering, South China University of Technology, Guangzhou, Guangdong, China; ^2^The Second People's Hospital of Shenzhen, Shenzhen, Guangdong, China; ^3^Shenzhen Institute of Geriatrics, Shenzhen, Guangdong, China; ^4^School of Mechanical Engineering, Guangxi University of Science and Technology, Liuzhou, Guangxi, China

## Abstract

The hemiplegic rehabilitation state diagnosing performed by therapists can be biased due to their subjective experience, which may deteriorate the rehabilitation effect. In order to improve this situation, a quantitative evaluation is proposed. Though many motion analysis systems are available, they are too complicated for practical application by therapists. In this paper, a method for detecting the motion of human lower limbs including all degrees of freedom (DOFs) via the inertial sensors is proposed, which permits analyzing the patient's motion ability. This method is applicable to arbitrary walking directions and tracks of persons under study, and its results are unbiased, as compared to therapist qualitative estimations. Using the simplified mathematical model of a human body, the rotation angles for each lower limb joint are calculated from the input signals acquired by the inertial sensors. Finally, the rotation angle versus joint displacement curves are constructed, and the estimated values of joint motion angle and motion ability are obtained. The experimental verification of the proposed motion detection and analysis method was performed, which proved that it can efficiently detect the differences between motion behaviors of disabled and healthy persons and provide a reliable quantitative evaluation of the rehabilitation state.

## 1. Introduction

Nowadays, the aging problem becomes a very topical and complicated social challenge [1, 2]. A high incidence and recurrence rate among aged people is exhibited by such disease as hemiplegia, which implies paralysis of one side of the body usually caused by a brain lesion, such as a tumor, or by stroke syndrome. The number of hemiplegia cases is increasing quickly, and a large share of survivors after stroke become disabled (about 70%) and even severely disabled (about 40%) [3].

The correct diagnosis of the motion disorders is critical for prescribing an effective treatment, but the judgment of therapists is based on their experience of therapists and, thus, is somewhat subjective. The unbiased representation of the patient state is the basic requirement for developing the best treatment matching this state and reducing the rehabilitation period.

Motion detection and parametric description are the main components of the integral evaluation system. Multiple detecting methods have been developed in the last decades, including WB-4 [4], Vicon [5], OPTOTRAK of Northern Digital [6], STAGE of Organic Motion [7], Kinect of Microsoft [8], Liberty 240/8 of Polhemus [9], NDI [10], and HX17 of Hexamite [11]. In view of such factors as the rehabilitation environment complexity, mechanical tracking having a complex calibration, optical sensors being interfered by therapists, low accuracy of the acoustic tracking, and electromagnetic tracking vulnerability to metal interference, many motion detection methods fail to meet the detection requirements.

The inertial sensing technology is a relatively innovative motion tracking system with high performance and large measurement range, which utilizes easy wearable portable inertial measurement units (IMUs). Recently, inertial sensor has been used to detect and evaluate human motion: whole body motion [12, 13], scapula calibration [14], lie-to-stand transfer [15], and gait analysis [16–20]. The studies using inertial sensors measure movement time; calculate joint or inclination angles, walking speed, step or stride length, and segment position relative to other position; and detect gait event timings. However, there are certain limitations on the applications, which impede more effective diagnosing and training as to the simplifying or resolving of the joint motion and motor function evaluation according to the clinical requirement of the therapist diagnosing.

This paper aims to detect the motion of human lower limbs via the inertial sensors, which permits analyzing the motion ability according to clinical rehabilitation needs. This method is applicable to arbitrary walking directions and tracks of persons under study, and its results are unbiased, as compared to therapist qualitative estimations. Using the simplified mathematical model of a human body, the rotation angles for each lower limb joint DOF are calculated from the input signals acquired by the inertial sensors via the respective gesture quaternion. Finally, the rotation angle versus joint displacement curves are constructed, and the estimated values of joint motion angle and motion ability are obtained. The experimental verification of the proposed motion detection and analysis method was performed, which proved that it can efficiently detect the differences between motion behaviors of disabled and healthy persons and provide a reliable quantitative evaluation of the rehabilitation state.

The rest of the paper is organized as follows.  Section 2 gives a detailed description of the proposed system configuration.  Section 3 presents the experimental results. The performance and potential improvements of the proposed system are discussed in Section 4.

## 2. Methods

### 2.1. System Overview

**Figure 1:**
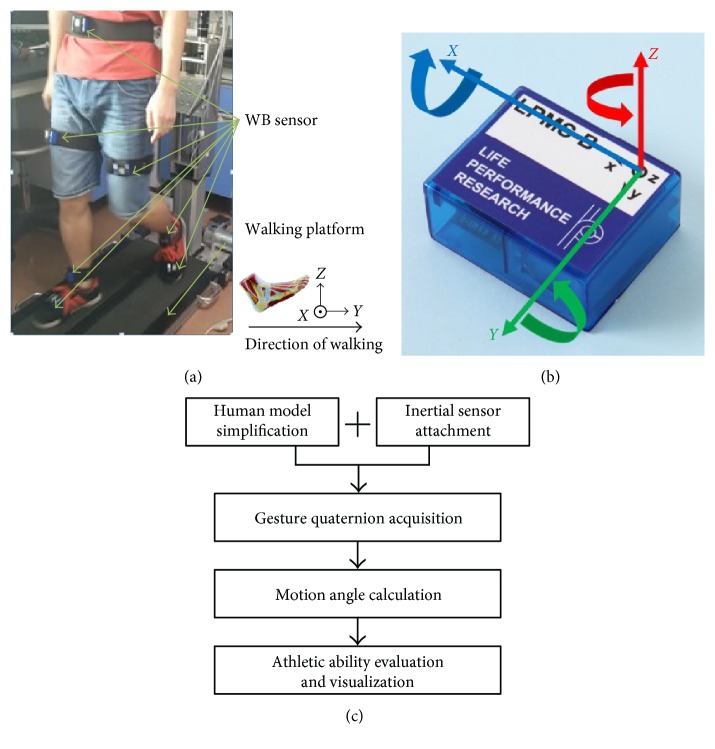
(a) Walking platform. (b) WB sensor. (c) System flowchart.

The system comprises a platform [21] for rehabilitation training (with weight support device and pelvic fixation device) as shown in Figure 1(a), seven WB sensors depicted in Figure 1(b) and a laptop with bespoke data processing software and a graphic user interface (GUI) developed in C++ Builder. The system aimed at acquiring the studied participant's gait kinematics indicated by hip knee and ankle angle of each joint's degree of freedom (DOF) and evaluating the athletic ability from the motion angle phase. This is achieved by acquiring the gesture quaternion of the studied participant from the inertial sensors attached to his/her waist, thigh, crus, and foot when he/she walks on the platform. As shown in Figure 1(c), the system procedure includes human model simplification and inertial sensor attachment, gesture quaternion acquisition, motion angle calculation, and athletic ability evaluation and visualization. Each of these acquisition and processing steps is described in the following.

### 2.2. Human Model Simplification and Inertial Sensor Attachment

**Figure 2:**
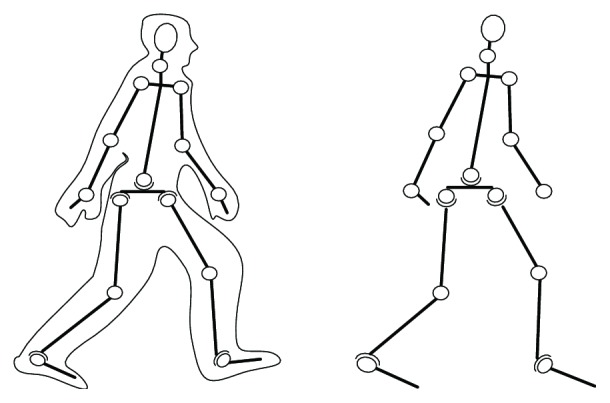
The simplified human motion model.

Human body skeleton has a very complex structure, which must be simplified, in order to achieve the real-time analysis of human motion, using special models of human body motion models. Thus, the essence of the stick figure model is that it reduces the human body motion to that of human skeleton bones, so that various parts of a human body are approximated by the straight lines. For example, the stick figure model proposed by Chen and Lee [22] contains 17 sections and 14 connection points to represent the head, torso, and limbs. Since the different models have different motion mathematical relationships, different models will lead different results by the same acquired data. The simplified human motion model used herein is depicted in Figure 2.

The human skeleton model used in this study regards a human skeleton as a rigid rod and reduces a knee to a uniaxial joint according to the clinical diagnosing requirement. The DOFs of hip and ankle are three, and the DOF of knee is only one.

Within the framework of the applied inertial sensing technology, which was briefly discussed in Section 1, the life performance research motion sensor (LPMS) was selected as the motion sensor. The LP-Research Motion Sensor Bluetooth version (LPMS-B) is a miniature wireless IMU/attitude and heading reference system (AHRS). The unit is very versatile, performing accurate, high-speed orientation, and displacement measurements. By the use of three different MEMS sensors (3-axis gyroscope, 3-axis accelerometer, and 3-axis magnetometer), drift-free, high-speed orientation data about all three axes is achieved. The LPMS-B communicates with a host system via a Bluetooth connection. The LPMS sensor measures the orientation difference between the fixed sensor and global reference coordinate systems.

(1) $$\begin{array}{c} Q_{sensor}=q_{difference}Q_{global}{q_{difference}}^{-1}, \end{array}$$

where *Q*_sensor_ is the fixed sensor coordinate system, *Q*_global_ is the global coordinate system, and *q*_difference_ is the orientation difference between *Q*_global_ and *Q*_sensor_.

**Figure 3:**
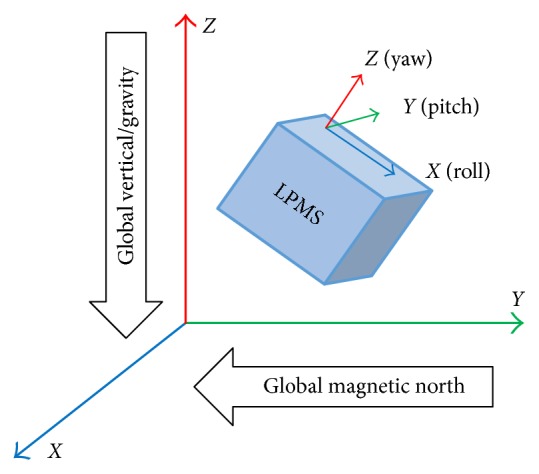
Global and local sensor coordinates.

The local and global reference coordinate systems used are defined as right-handed Cartesian coordinate systems, where *X* is positive when pointing to the magnetic West, *Y* is positive when pointing to the magnetic South, and *Z* is positive when pointing up (in the opposite direction to gravity vector). The axial orientation of LPMS-B and the relationship between the local sensor coordinate system and global coordinates are shown in Figure 3.

### 2.3. Gesture Quaternion Acquisition

This section includes the definition of quaternion and discusses its acquisition procedure.

There are various ways of representing orientation, and the use of Euler angles is one of them. The Euler angles are used to represent roll, pitch, and yaw of a body. There is one constraint when using the Euler angles for this purpose: this representation has singularities at pitch angles of ±90°. Quaternions avoid these singularities by having a fourth element. The addition of this element is at the constraint of being a unit length. The concept of quaternion has been introduced by Hamilton in 1843 [23]. A special subset of the quaternion space, denoted by **IH**, is defined that when ||*q*|| = 1, then *q* is called a unit norm quaternion, and the unit quaternion space is denoted as **IH**_1_. This particular subset is of special interest, since it provides the characterization of orientation trajectories. The temporal orientation trajectories are studied in the unit norm quaternion space **IH**_1_ ⊂ **IH** [24]. Any general three-dimensional rotation can be transformed into a unit norm quaternion q ∈ **IH**_1_.

In this study, the rotation quaternion is expressed as *q* = (*w*, *x*, *y*, *z*)^*T*^, where *w* is the cosine of the rotation semiangle, while *x*, *y*, *z* are the multiplication X, Y, Z coordinates of the rotation axis and the sine of the rotation semiangle, whereas *w*^2^ + *x*^2^ + *y*^2^ + *z*^2^ = 1.

**Figure 4:**
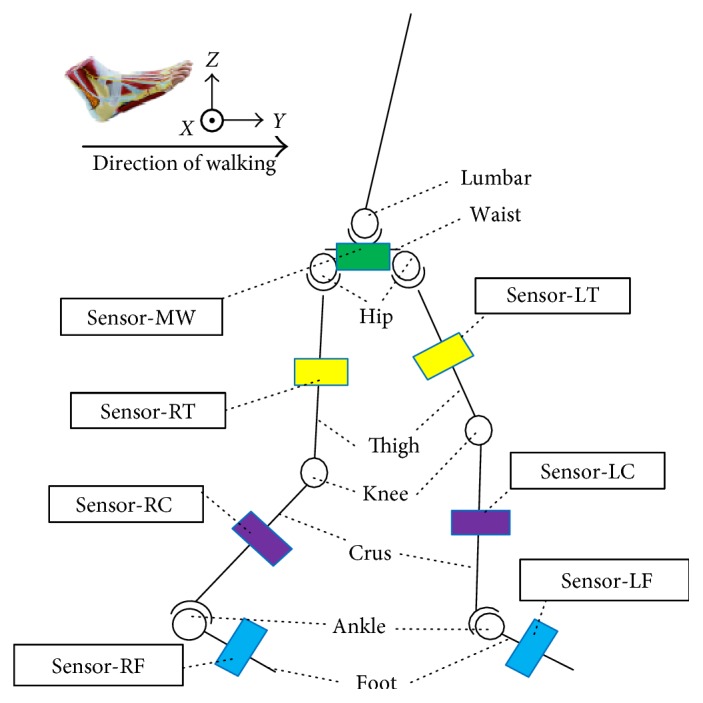
Location of attached sensors.

In order to acquire quaternions via the gesture quaternion recording technique, seven sensors are attached by Velcro straps to the waist, thigh, crus, and foot on both legs of the studied participant in the walking platform. The location and the orientation of sensors are shown in Figure 4, where the following designations are used: MW corresponds to the waist-attached sensor, while LT/LC/LF and RT/RC/RF are sensors attached to the left and right thigh/crus/foot, respectively.

**Figure 5:**
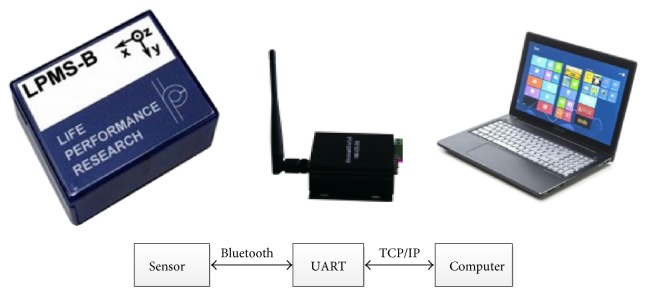
Hardware communication.

The hardware communication between sensors and PC is depicted in Figure 5. Here, the transceiver communicates with sensors via Bluetooth, obtains the sensor code key and MAC address, and then converts the latter to an IP address and port. The PC is connected with UART through the network communication and, thus, communicates with sensors attached to the specified parts of human body and acquires the gesture quaternion from them in the real-time scale.

### 2.4. Motion Angle Calculation

This section includes two parts: (1) mathematical modeling and (2) motion angle calculation according to the mathematical model and gesture quaternion.

1. Mathematical Modeling

It is necessary to elaborate the appropriate diagnostic criteria for the human lower limb joints, in particular, the left and right hips, knees, and ankles (six joints in total), which have different kinematics, motion functions, and range. A lot of efforts have been made to meet the needs of medical diagnosis and convenient modeling and measurement, resulting in the application of six coordinate systems corresponding to each joint and accounting for their structural and functional specifics.

**Figure 6:**
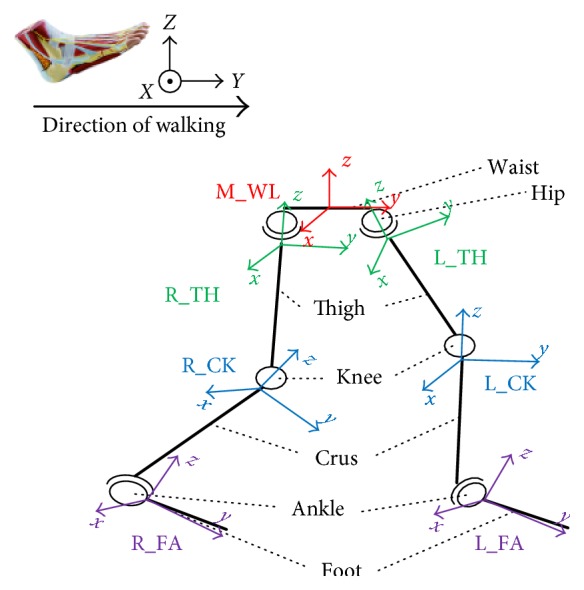
The mathematical model of the human lower limbs.

From the conventional medical standpoint, the hip athletic ability is assessed from the posture between the waist and thigh, the knee ability assessment is based on the relationship of thigh and crus, and the ankle is related to the crus and foot. In this paper, the analysis of the abilities of human lower limb is based on the same method as that used by the conventional medical approach. The mathematical model for lower limb motion detection is presented in Figure 6, where seven local coordinate systems are used, including the simplified waist level, left/right hip, left/right knee, and left/right ankle ones related to the gesture quaternion data acquired from the respective sensors, whose location is specified in Figure 4.

**Figure 7:**
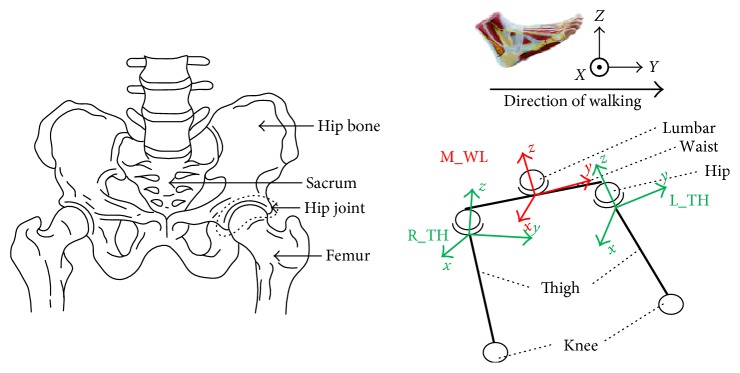
The physical structure and the coordinate system of the hip joint.

The human hip joint motion can be reduced to 3 DOFs: flexion-extension, exhibition-adduction, and internal-external rotation motion. Normally, the hip motion is compensatory. The range of hip motion is an important parameter for the human motion ability analysis. To analyze the ability of the hip, normally, three orthogonal axes are selected as the basic ones.  Figure 7 shows the simplified human structure with the coordinates. Here, taking the same roll of human body, the pitch axis is defined between the femoral ends, the row axis coincides with the femoral bone axis, the waist coordinates are depicted by M_WL, and the respective coordinate axes *x*, *y*, and *z* are drawn in red. The coordinate systems of the left and right thighs are depicted as L_TH and R_TH, respectively.

**Figure 8:**
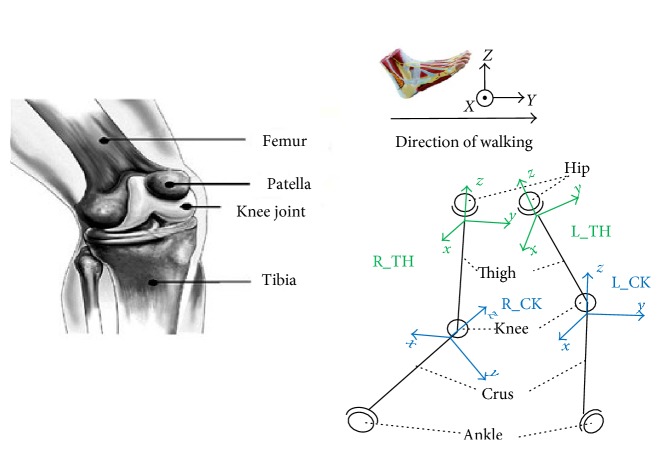
The physical structure and the coordinate system of the knee joint.

The knee joint plays a critical role in the human walking process. Its rotation angle range is 0 to 135 degrees. For the knee joint, the bending of the knee during walking is referred to as flexion/extension, while its rotation about the other two axes (abduction/adduction and internal/external rotation) is generally quite small for this joint. Therefore, the knee motion during walking can be reduced to one flexion-extension DOF. The original coordinates are matched with the femoral bone, human roll axis, and the other axis following the right-hand rule, as is shown in Figure 8, where the left and right crus coordinates are L_CK and R_CK, respectively.

**Figure 9:**
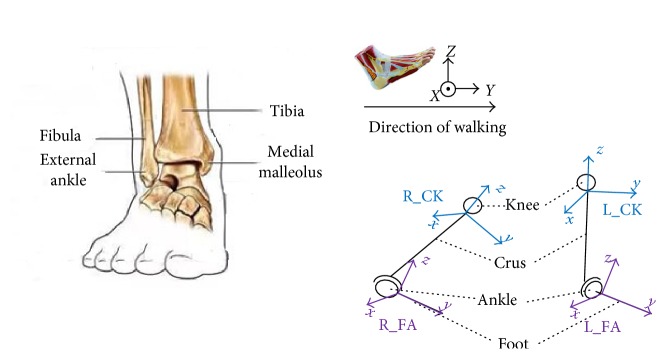
The physical structure and the coordinate system of the ankle joint.

Ankles are critical for balance-keeping in the walking process by realizing such foot actions like dorsiflexion, plantar flexion, abduction, adduction, and various eversions. As shown in Figure 9, the original coordinates coincide with the crus bone, human roll axis, and the other axis following the right-hand rule. The left and right ankle coordinates L_CK and R_CK, respectively, are acquired during motion together with the left and right feet ones—L_FA and R_FA, respectively.

Thus, the hip joint has three rotation DOFs about the *x*-, *y*-, and *z*-axes, the knee joint has one rotation DOF about *x*-axis, and the ankle joint has three rotation DOFs about the *x*-, *y*-, and *z*-axes. Each DOF can be reduced to a single quaternion angle value measured via two coordinate systems. So each lower limb motion is described by 7 angular measurements, which implies that 14 ones are required for the motion description of both lower limbs. The angle calculation method is as follows.

(2) *Motion Angle Calculation*

**Figure 10:**
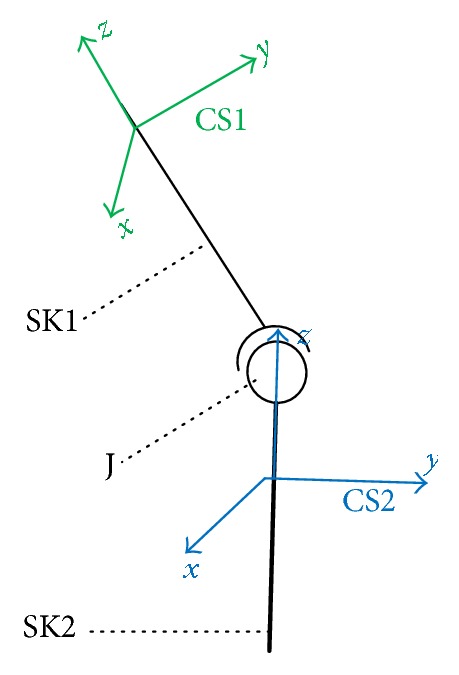
Mathematical model of the joint.

A mathematical model of joint is shown in Figure 10, where SK1 and SK2 stand for two bones connected by the joint *J*, and their coordinate systems are CS1 and CS2.

Two quaternions are acquired from sensors adjacent to the joint *J*. The SK1 gesture quaternion is defined as *q*_*J*SK_*n*__ and that of K2 as *q*_*J*SK_*n*+1__. Then, quaternion data have to be calibrated by their initial quaternion values.

(2) $$\begin{array}{c} q_{J_{n}}=q_{{JSK}_{n}}*{q_{J{SK0}_{n}}}^{-1}\\ q_{J_{n+1}}=q_{J{SK}_{n+1}}*{q_{JSK0_{n+1}}}^{-1}, \end{array}$$

where *q*_*J*SK0_*n*__ and *q*_*J*SK0_*n*+1__ are the initial quaternions when the patient/participant under study stands still, while *q*_*J*_*n*__ and *q*_*J*_*n*+1__ are the respective quaternions after calibration.

Then, a conversion quaternion *q*_*JC*_ can be calculated by the following equation:

(3) $$\begin{array}{c} {\;q}_{J_{n+1}}*{q_{J_{n}}}^{-1}. \end{array}$$

Defining the unit vector about *x*-, *y*-, and *z*-axes of the coordinate system by *V*_*JCU*_, the unit vectors after conversion *V*_*JCU*_*x*__′, *V*_*JCU*_*y*__′, and *V*_*JCU*_*z*__′ can be calculated via

(4) $$\begin{array}{c} {V_{JCU}}^{\prime }=q_{JC}*V_{JCU}*{q_{JC}}^{-1}, \end{array}$$

where

(5) $$\begin{array}{c} V_{JCU}=\left[V_{{JCU}_{x}},V_{{JCU}_{y}},V_{{JCU}_{z}}\right], \end{array}$$

(6) $$\begin{array}{c} V_{JCU}=\left[V_{{JCU}_{x}},V_{{JCU}_{y}},V_{{JCU}_{z}}\right]=\left[\begin{array}{ccc} 1 & 0 & 0\\ 0 & 1 & 0\\ 0 & 0 & 1 \end{array}\right]. \end{array}$$

 *V*_*JCU*_*x*__,  *V*_*JCU*_*y*__, and *V*_*JCU*_*z*__ are the unit vectors about the three axes.

Next, a vector projected by the unit vector rotated by conversion quaternion can be calculated via

(7) $$\begin{array}{c} P_{{JC}_{TM}}=M_{P_{TM}}*{V_{JCU}}^{\prime }*{M_{C}}_{TM}, \end{array}$$

where TM corresponds to the *x*-, *y*-, and *z*-axes and *P*_*JC*_*x*__, *P*_*JC*_*y*__, and *P*_*JC*_*z*__ are vectors projected on YOZ, XOZ, and XOY planes, respectively; and

(8) $$\begin{array}{c} \begin{array}{c} M_{P_{x}}=\left[\begin{array}{ccc} 0 & 0 & 0\\ 0 & 1 & 0\\ 0 & 0 & 1 \end{array}\right],\\ M_{P_{y}}=\left[\begin{array}{ccc} 1 & 0 & 0\\ 0 & 0 & 0\\ 0 & 0 & 1 \end{array}\right],\\ M_{P_{z}}=\left[\begin{array}{ccc} 1 & 0 & 0\\ 0 & 1 & 0\\ 0 & 0 & 0 \end{array}\right], \end{array} \end{array}$$

(9) $$\begin{array}{c} \left[M_{C_{x}},M_{C_{y}},M_{C_{z}}\right]=\left[\begin{array}{ccc} 0 & 0 & 1\\ 1 & 0 & 0\\ 0 & 1 & 0 \end{array}\right]. \end{array}$$

They are used to operate the matrix by simple row and column transformations.

The rotation angle about the coordinate axis can be calculated via

(10) $$\begin{array}{c} \theta_{J_{TM}}={atan2}_{TM}\langle P_{{JC}_{TM}},M_{C_{TM}}\rangle , \end{array}$$

where *θ*_*J*_TM__ is the rotation angle about the TM axis. Function atan2_*x*_(*A*, *B*) returns the argument of plural (*y*_*B*_ + *z*_*A*_*i*) of two three-dimensional coordinates A(*x*_*A*_, *y*_*A*_, *z*_*A*_) and *B*(*x*_*B*_, *y*_*B*_, *z*_*B*_); atan2_*y*_(*A*, *B*) returns the argument of plural (*z*_*B*_ + *x*_*A*_*i*) of two three-dimensional coordinates *A*(*x*_*A*_, *y*_*A*_, *z*_*A*_) and *B*(*x*_*B*_, *y*_*B*_, *z*_*B*_); and atan2_*z*_(*A*, *B*) returns the argument of plural (*x*_*B*_ + *y*_*A*_*i*) of two three-dimensional coordinates *A*(*x*_*A*_, *y*_*A*_, *z*_*A*_) and *B*(*x*_*B*_, *y*_*B*_, *z*_*B*_).

The angular speed and acceleration can be calculated by taking derivative of the rotation angle.

(11) $$\begin{array}{c} \begin{array}{c} \omega_{J_{TM}}=\frac{d\theta_{J_{TM}}}{dt}\\ \alpha_{J_{TM}}=\frac{d\omega_{J_{TM}}}{dt}, \end{array} \end{array}$$

where *ω*_*J*_TM__ is the rotation angular speed about the TM axis and *α*_*J*_TM__ is the rotation angular acceleration about the TM axis.

The joint *J* includes left hip (LP), left knee (LK), left ankle (LA), right hip (RH), right knee (RK), and right ankle (RA). Here, *n* is the number of sensors ranging from 1 to 7.

### 2.5. Athletic Ability Evaluation and Visualization

The joint angle graph describes the relationship between the angular variations of each joint in the gait cycle for the total gait cycle phase [25, 26]. The patients with hemiplegia exhibit the unilateral lower limb symptoms, such as inability of exercising one lower limb or unilateral handicap obstructing their harmonious motion, which are reflected in their lower limb rotation angle-angular speed curves. The difference and correlation between the above curves constructed for different joints of healthy and disabled participants can reflect the degree of the patient illness, if any. By calculating the residual sum of squares for the motion data of the lower limb target side, as compared to those measured for the other side, the related ability degrees of healthy and disabled participants can be estimated.

(12) $$\begin{array}{c} R_{J}=\overset{7}{\underset{i=1}{\sum }}\rho_{i}R_{i}, \end{array}$$

where *R*_*J*_ is the rehabilitation evaluation of joint, *R*_*i*_ is the rehabilitation degree of each DOF of joint, and *ρ*_*i*_ is the adjustment coefficient of *R*_*i*_. Since there are 7 motion angles, *ρ* = [0.3 0.15 0.15 0.28 0.06 0.03 0.03].

The joint motion angular speed is a key parameter to reflect the performance of joint athletic ability, and the rotation angle-angular speed curve can provide a judgment for the joint motion characteristics.

(13) $$\begin{array}{c} E_{J}=\overset{7}{\underset{i=1}{\sum }}\frac{A_{Di}}{A_{Hi}}, \end{array}$$

where *E*_*J*_ is the rehabilitation state estimate: the closer to 1, the better rehabilitation state. *A*_*Di*_ and *A*_*Hi*_ are the track areas of lower limb rotation angle-angular speed curves of disabled and healthy participants, respectively.

## 3. Results

**Figure 11:**
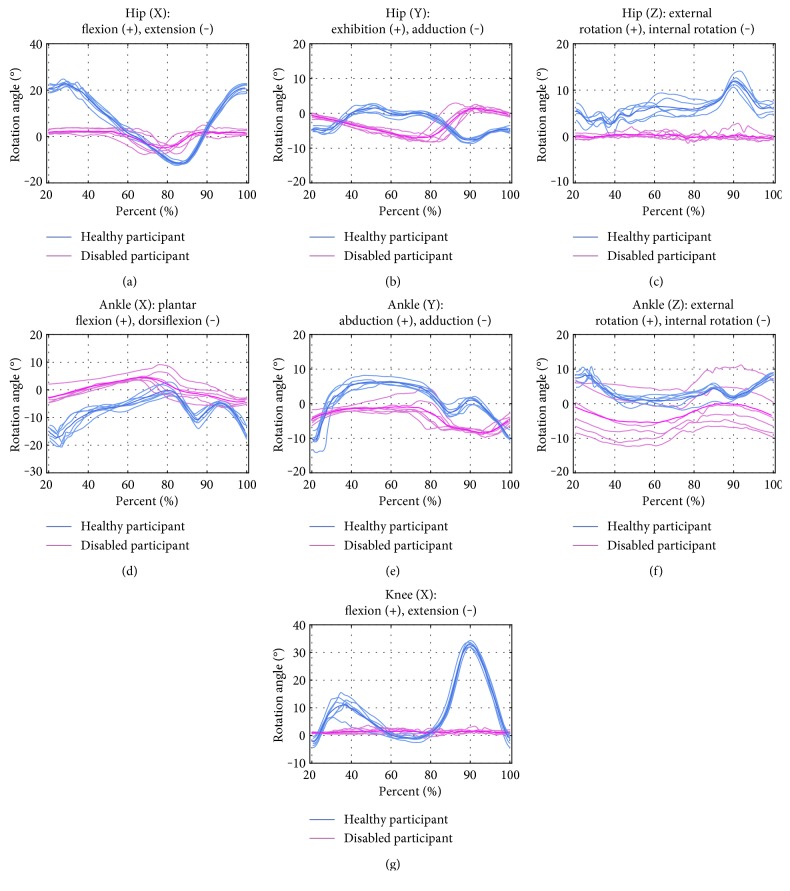
Rotation angle curves of joints' motion: 3 DOFs for hip (a, b, c), 3DOFs for ankle (d, e, f), and 1 DOF for knee (g). Here and in Figure 12, curves of healthy and disabled participants are shown in blue and red, respectively.

**Figure 12:**
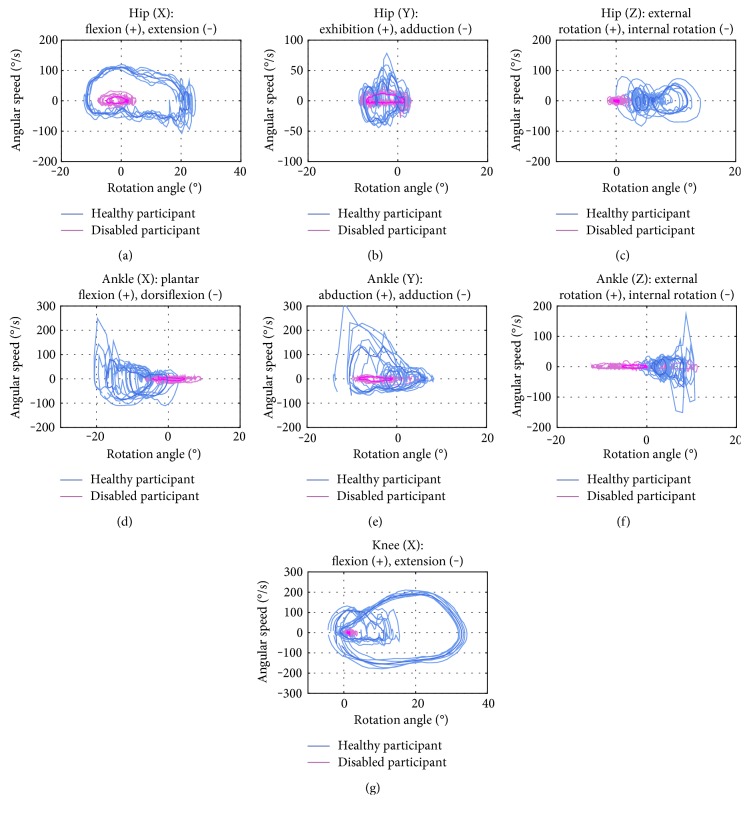
Rotation angle-angular speed curves of joints: 3 DOFs for hip (a, b, c), 3 DOFs for ankle (d, e, f), and 1 DOF for knee (g).

A series of walking experiments have been conducted to get the motion data of disabled and healthy participants. The disabled participant, 43 years old, has a serious movement dysfunction on his left side since the sequel of cerebrovascular disease. He suffers from strephenopodia on the ill side. His knee joint cannot bend normally, and he has to use his waist muscle making up the hip joint instead of the ankle flexion, so that he can lift toe off the ground and complete the step. The motion angle curves of disabled and healthy participants' hips knees and ankles are shown in Figure 11. The evaluation of disabled participant's joint motion angles is made via Equation (12), where *R*_*J*_ = 0.2964. The rotation angle versus angular speed curves of disabled and healthy participants' lower limbs are shown in Figure 12. The evaluation of rehabilitation state of the disabled participant is performed via Equation (13), where *E*_*J*_ = 0.1951. The obtained value of *R*_*J*_ implies the large differences between the disabled and healthy participant's lower limb athletic abilities.

The disabled participant has a serious motion dysfunction, as compared with the healthy one, as is shown in Figure 11 and indicated by the obtained value of *R*_*J*_ = 0.2964. The disabled participant's hip, knee, and ankle DOFs about the *x*-axis are limited and cannot accomplish the whole gait cycle properly, while the hip DOF about the *z*-axis is close to zero, while the ankle DOF about the *z*-axis exhibits a constant difference from that of the healthy participant.

The resulting disabled participant rehabilitation state estimate is not quite optimistic as is shown in Figure 12 and indicated by the obtained value of *E*_*J*_ = 0.1951. The athletic ability of joints, such as hip joint about the *x*- and *y*-axes, knee joint about the *x*-axis, and ankle joint about the *x*- and *y*-axes, is so deteriorated that a long rehabilitation period is required. The athletic ability of both hip and ankle joints about the *z*-axis exhibits a serious morbidity, insofar as the track area of rotation angle versus angular speed curves is far from that of the healthy participant.

The experimental results obtained imply that the proposed inertial sensor-based method of the lower limb motion analysis is quite practical, reliable, and applicable for rehabilitation state evaluation. The gesture quaternion of lower limbs based on inertial sensors can be converted to angles. The joint rotation angle can be calculated using the simplified lower limb motion model. Finally, the rotation angle versus angular speed curves for the hips, knees, and ankles are constructed using the proposed algorithm, and the analysis of joint motion angles and athletic ability is provided, in order to evaluate the rehabilitation state.

## 4. Conclusions and Future Work

Gait motion analysis plays an important role in the patient state evaluation. In this paper, a method for detecting the motion of human lower limbs including all degrees of freedom via the inertial sensors is proposed, which permits analyzing the motion ability according to the rehabilitation needs. This method is applicable to arbitrary walking directions and tracks of persons under study, and its results are unbiased, as compared to therapist qualitative estimations. Using the simplified mathematical model of a human body, the rotation angles for each lower limb joint are calculated from the input signals acquired by the inertial sensors via the respective gesture quaternion. Finally, the rotation angle versus joint displacement curves are constructed, and the estimated values of joint motion angle and motion ability are obtained. The experimental verification of the proposed motion detection and analysis method was performed, which proved that it can efficiently detect the differences between motion behaviors of disabled and healthy persons and provide a reliable quantitative evaluation of the rehabilitation state.

As a future work, the proposed model refinement and more fine calibration of the experimental setup for minimization of motion detection errors are envisaged, in order to improve the method effectiveness and functionality. Next, the applied lower limb motion detection method can be integrated into the rehabilitation robot control system, realizing intelligent detection and evaluation. Eventually, the rehabilitation robots can be elaborated, which would provide the automatic adjustment of training parameters based on the particular patient status. Upon incorporation of the above features into the system, experiments will be arranged among hemiplegic patients to verify the feasibility and efficiency of the motion detection, robot control, and rehabilitation evaluation systems. Further development of this research is expected to have a significant influence on the motion detection, rehabilitation evaluation, and medical rehabilitation robot domains.
